# Biocatalysts Based on Immobilized Lipases for the Production of Fatty Acid Ethyl Esters: Enhancement of Activity through Ionic Additives and Ion Exchange Supports

**DOI:** 10.3390/biotech12040067

**Published:** 2023-12-18

**Authors:** Juan S. Pardo-Tamayo, Sebastián Arteaga-Collazos, Laura C. Domínguez-Hoyos, César A. Godoy

**Affiliations:** Laboratorio de Investigación en Biocatálisis y Biotransformaciones (LIBB), Grupo de Investigación en Ingeniería de los Procesos Agroalimentarios y Biotecnológicos (GIPAB), Departamento de Química, Universidad del Valle, Cali 760042, Colombialaura.camila.dominguez@correounivalle.edu.co (L.C.D.-H.)

**Keywords:** enzyme immobilization, biodiesel, ionic additives, lipase activation, lipase derivatives

## Abstract

Ionic additives affect the structure, activity and stability of lipases, which allow for solving common application challenges, such as preventing the formation of protein aggregates or strengthening enzyme–support binding, preventing their desorption in organic media. This work aimed to design a biocatalyst, based on lipase improved by the addition of ionic additives, applicable in the production of ethyl esters of fatty acids (EE). Industrial enzymes from *Thermomyces lanuginosus* (TLL), *Rhizomucor miehei* (RML), *Candida antárctica B* (CALB) and Lecitase^®^, immobilized in commercial supports like Lewatit^®^, Purolite^®^ and Q-Sepharose^®^, were tested. The best combination was achieved by immobilizing lipase TLL onto Q-Sepharose^®^ as it surpassed, in terms of %EE (70.1%), the commercial biocatalyst Novozyme^®^ 435 (52.7%) and was similar to that of Lipozyme TL IM (71.3%). Hence, the impact of ionic additives like polymers and surfactants on both free and immobilized TLL on Q-Sepharose^®^ was assessed. It was observed that, when immobilized, in the presence of sodium dodecyl sulfate (SDS), the TLL derivative exhibited a significantly higher activity, with a 93-fold increase (1.02 IU), compared to the free enzyme under identical conditions (0.011 IU). In fatty acids ethyl esters synthesis, Q-SDS-TLL novel derivatives achieved results similar to commercial biocatalysts using up to ~82 times less enzyme (1 mg/g). This creates an opportunity to develop biocatalysts with reduced enzyme consumption, a factor often associated with higher production costs. Such advancements would ease their integration into the biodiesel industry, fostering a greener production approach compared to conventional methods.

## 1. Introduction

Biocatalysis has been widely used at an industrial level (white biotechnology) due to its advantages in terms of specificity, mild reaction conditions and as a green alternative to the conventional catalysts—highly polluting and toxic solvents or reagents—resulting in the intensification and higher sustainability of the processes [1,2,3]. An example of this occurs in the energy sector, which has benefited from lipase-based biocatalyst implementation for the production of fatty acid alkyl esters (EE) from vegetable oils, which are the main component of biodiesel [4,5,6]. This is because the use of lipase-based biocatalysts reduces the use of strongly oxidizing/corrosive agents like sulfuric acid or potassium hydroxide. Furthermore, this enables the use of lower-quality raw materials, reducing the purification steps currently required for the conventional industrial process [4,6,7,8].

For this reason, in the production of EE using biocatalysts, it is not necessary to use oils/fats which should be prioritized for human consumption, opening up the possibility of exploiting oils/fats that are often the typical waste of industrial and domestic practices [9,10]. Furthermore, these wastes could ultimately lead to contamination of vast quantities of water (1 L can contaminate 40,000 L of water) [9,11,12]. Thus, implementing biocatalysis in the biodiesel industry would add value to waste oils, which would achieve an approximation of the circular economy ideal [13].

Lipases are the third most commercialized enzymes, after proteases and carbohydratases, and are applied to the industrial sector for their wide range of catalyzing reactions and the substrates that accept them. Additionally, engineering tools are available to optimize their stability and activity under the required operating conditions [14,15]. In particular, microbial lipases are the most used because, due to the adaptive processes of the organisms of origin, a wide range of working conditions are available (pH, temperature, ionic strength) [16]; they are typically active as monomers and do not need colipases [17]. Moreover, due to their extensive research, microbial lipases have the possibility for large-scale production through strategies like recombinant DNA technology. This enables the convenient and cost-effective extraction of lipases from complex systems or even utilizing wild crops [18].

Focusing on the production of EE, it has been shown that short-chain alcohols, used as raw materials, induce conformational changes that lead to protein aggregation and, thus, their deactivation [19,20]. Lipase immobilization on mesoporous supports has been investigated as a tool to mitigate such limitations [19,21]. One of the most used commercial immobilized lipases is Novozyme^®^ 435 (CALB immobilized on Lewatit^®^ VPOC1600). Due to the hydrophobic characteristic of the support, alcohol can induce immobilized lipase aggregation and modify the support texture, generating desorption of the enzyme, thus decreasing biocatalyst activity [19]. Therefore, there is still the need to find new strategies to circumvent this hurdle.

Some lipases present conformational equilibrium [22,23]. In an aqueous environment, they are typically in a closed, inactive form with a minority in an open, active state due to the positioning of the lid [22,23]. The equilibrium changes in the presence of water–lipid interfaces, leading to the interfacial activation in some lipases [22,24]. Creating environments that stabilize the open conformation enhances catalytic activity, known as “hyperactivation” [25,26]. An illustrative case of this phenomenon can be seen in numerous instances involving free or immobilized lipases exposed to ionic or non-ionic surfactants in aqueous solutions. It has been concluded that the hydrophobic part of the surfactant interacts with the hydrophobic active site of the lipase, while the hydrophilic part interacts with the medium, promoting its active form [27,28]. However, the extent of the hyperactivation depends on the nature of the lipase, surfactant, reaction medium and the substrate involved [29]. These surfactants not only impact lipase activity but also influence their selectivity (substrate preference), and may serve as spacer arms connecting a support material and the lipase itself [25,27]. Thus, it is necessary to collect data on the effects that ionic additives may have on lipases, which allow us to understand and model the interactions they generate for the design of lipase immobilizations focused on obtaining highly efficient and operationally stable biocatalysts [25]. This requires tuning critical parameters of the enzyme immobilization process, such as medium conditions (pH, ionic additives, immobilization time, ionic strength, etc.) and support characteristics (structure, pore size, etc.) [24,25].

Thus, in this research, obtaining immobilized lipases, also known as lipase derivatives, for the production of fatty acid alkyl esters (EE) was attempted from the immobilization of commercial lipases which are typically used in the biodiesel industry, such as those from *Thermomyces lanuginosus* (TLL), *Rhizomucor miehei* (RML), *Candida antarctica* B (CALB) and Lecitase^®^ [6,7,21], on commercial supports such as Lewatit^®^ VPOC1600, Q-Sepharose^®^ and Sulfopropyl Sepharose^®^, among others [21,30]. In that context, we also included the use of ionic additives that modulated lipases’ properties to obtain more efficient and potentially scalable biocatalysts for the biodiesel industry.

## 2. Materials and Methods

### 2.1. Materials

CAL B, TLL, RML, Lecitase^®^, octyl-Sepharose^®^, sulfopropyl-Sepharose^®^, Q-Sepharose^®^, *p*-nitrophenyl butyrate (*p*-NPB), Cetyltrimethylammonium bromide (CTAB), Ethylenediaminetetraacetic acid (EDTA), Bicinchoninic Acid Kit (BCA), Bovine serum albumin (BSA), ethanol (96%) and salts for buffering solutions were purchased from Sigma Chem. Co. (St. Louis, MO, USA). Nekrolith^®^ support was purchased from Mitsubishi Chemical. Other supports, PEI-agarose and DexSO_4_-agarose, were prepared according to Guisan [31,32]. Palm olein was purchased in a local store. Unrefined palm oil and used cooking oil were donated by Biocombustibles Sostenibles del Caribe S.A. (Ing. Carlos Velásquez) and Redciclar (Foundation Crese), respectively. Novozyme^®^ 435 and Lipozyme^®^ TL IM (commercial biocatalysts based on immobilized CALB and TLL) was a gift from Novozymes (Bagsværd, Denmark). Other reagents and solvents were of analytical or HPLC grade. Supports from Lewatite^®^ (VP OC 1600 (LW), based on polymethacrylate/divinylbenzene copolymer, and MP 800 (MP), based on cross-linked polystyrene functionalized with Type I quaternary ammonium groups, were kindly donated by Lanxess^®^ (Cologne, Germany) and Purolite^®^ ECR1604 (PU), based on based on polymethacrylate/divinylbenzene copolymer functionalized with Type I quaternary ammonium groups, was donated by Purolite Ltd. (Llantrisant, UK).

### 2.2. Esterase Activity and Protein Determination

The esterase activity of soluble or immobilized enzymes against *p*-NPB (p-nitrophenyl butyrate) was assayed at pH 7.0 (25 mM sodium phosphate buffer) and 25 °C as previously described [33], with the following modifications: presence of Triton^®^ X-100 (TX) 0.01% for CALB or RML and CTAB 0.001% and TX 0.01% for TLL. In experiments involving the variation of surfactant concentrations, the amounts of the surfactants were adjusted correspondingly. One international unit (IU) is defined as the amount of enzyme required to hydrolyze one μmol of *p*-NPB min^−1^ under the conditions described above.

Protein determination was performed according to the Protein Assay Kit protocol at 37 °C for 30 min using BSA (bovine serum albumin) as a standard (Pierce^®^ BCA). The quantity of protein was measured in proper dilutions of filtered aliquots of control and immobilization supernatants after the decantation of the support. The protein loading on the different immobilization supports was calculated from the difference in protein content measured between the control and the respective immobilization supernatant after 24 h [34].

### 2.3. Immobilization of Lipases: Obtaining Lipase Derivatives

The production of highly loaded immobilized lipases (derivatives) was performed by mixing 1.00 g of support and 40.0 mL of a solution with 2 mM EDTA, 10% glycerol, with a protein concentration of 1.86 mg/mL and 10.0 mM buffer (sodium phosphate or citrate) for 24 h at 28 °C and at the desired pH. Once TLL and Q-Sepharose^®^ were selected as the best combination, CTAB (0.005%) or SDS (0.1%) were added to the immobilization solution and the protein concentration was varied in order to produce derivatives with different enzyme loads. Subsequently, the derivatives were concomitantly washed with immobilization solution without enzyme and then with distilled water. Finally, the derivatives were stored at 4 °C until use.

### 2.4. One-Step Solvent-Free Fatty Acid Ethyl Ester Production (EE)

A mass of 40 mg of selected lipase derivatives (wet basis, equivalent to 6% of the oil mass) was added to 1.17 g of palm olein and ~190 mg of absolute ethanol (ethanol:oil molar ratio 3.1:1), without any solvents or additives. These components were combined in hermetic vials, which were then placed on a Thermomixer^®^ at 37 °C and 1700 rpm. Samples of 50 μL were withdrawn at different times, and the content of fatty acid ethyl esters (EE) was analyzed using FTIR-ATR spectroscopy [9].

For reuse of TLL- SDS-Q derivatives, at the end of the reaction time (6 h) the oil phase was extracted and the biocatalyst washed with 350 μL of 0.1% SDS twice. Finally, the derivative was washed with 10 mM phosphate buffer at pH 7.0 thrice and used in a new reaction cycle.

### 2.5. Spectroscopic Measurements of Derivatives and Supports

For FTIR-ATR measurements, the derivatives or supports (0.2 g) were washed ten times with 10 mL immobilization solution without additives and enzymes and then with deionized water, then filtered and dried at 30 °C under vacuum overnight until obtaining constant mass. FTIR-ATR spectra of the dried derivatives or supports were recorded using a Perkin Elmer Spectrum (with the Spectrum^TM^ Software) from 600–4000 cm^−1^ with 25 scans and 4 cm^−1^ resolution and an ATR probe with a cleaned diamond 3-reflection plate at the highest pressure for the Clamp (Pike Miracle^TM^ technologies). Normalization and ATR correction were performed using the Spectrum^TM^ Software [35].

### 2.6. SDS-PAGE Characterization of the Biocatalysts

SDS-PAGE experiments were carried out following protocols previously reported by Javier Rocha-Martin et al. [36], with some modifications. The derivatives’ samples were diluted in 4% SDS (*w*/*v*) and 10% mercaptoethanol (*v*/*v*) to have a protein concentration of 0.5 mg of protein/mL solution in the samples. Also, for the ionic derivatives alone, NaCl was used to reach 1.0 M. The sample solutions were boiled for 10 min. The support was discarded after centrifuging the suspension at 4000 rpm for 2 min. After taking 15 μL aliquots of the supernatants of each sample and 7 μL of LMW-SDS Marker BioRad #1610374 (10–250 kDa), the samples were injected in 12% polyacrylamide gels, which were run at 100 V. Proteins were stained using Coomassie blue dye.

### 2.7. TLL-Additive In Silico Modeling

For the modeling of the enzyme–additive coupling, the docking software, PyRx, using AutoDock 4 and AutoDock Vina, was used (http://pyrx.sourceforge.net, The Scripps Research Institute, accessed on 17 May 2023) [37]. Three-dimensional crystalline structures of open TLL (PDB ID = 6XOK) were downloaded from the Protein Data Bank (PDB, http://www.rcsb.org/pdb/, accessed on 14 April 2023). Three-dimensional structures for additives were obtained by converting MDL SDfiles to PDB files, using the SMILES online structure generator and translator (https://cactus.nci.nih.gov/translate/, National Cancer Institute/Chemical Biology Laboratory, accessed on 13 April 2023). In order to facilitate the calculations, in the case of the polymers PEI and CMC, we decided to evaluate their coupling using a representative oligomer of each (*n* = 29 for PEI; *n* = 7 for CMC with substitution degree 0.7). The X, Y and Z coordinates of the grid center for the point of binding of the additives were fixed over the entire enzyme area (X = 547,487; Y = 314,687; Z = 820,317). Models with the highest free binding energy (lower coupling energy), ΔG, were chosen to explore the additive’s position with respect to the enzyme via visualizing docking results in open-access PyMOL software (https://pymol.org/2/, accessed on 17 May 2023).

### 2.8. Statistical Analysis

The experiments described were performed in triplicate. An ANOVA procedure (*p* < 0.05) was used to evaluate significant differences among means.

## 3. Results and Discussion

### 3.1. Reversible Immobilization of Lipases in Different Types of Commercial Supports

The results of the immobilization of the commercial lipases TLL, RML, CALB and Lecitase^®^, on different the commercial supports of reversible immobilization are summarized in Table 1.

According to the above results, the Lewatit^®^ VPOC 1600 support had an immobilized activity performance above 82% for all lipases used, due to the natural affinity of this type of enzyme to lipid–water interfaces [22,24,41]. In addition, it can be inferred that immobilization via ion exchange interactions is also high (up to 94.1% for Lewatit^®^ MP800), given that the quantity of the ionic groups on the enzyme’s surface is lower compared to the hydrophobic groups (Figure S1), especially in the close lipase conformation; this phenomenon could be attributed to the increased strength of the ionic protein–support interactions in comparison to the hydrophobic protein–support interactions [42]. In addition, a greater dispersion of the ionic groups was observed, favoring the anchoring of the enzyme to the support at a greater variety of angles, enabling a multipoint union with the activated groups and the appropriate protein–support coupling [43]. CALB obtained the lowest percentages of immobilization on anion exchange supports, presumably because it has the highest pI among the lipases studied here (8.12) in addition to the most balanced amount of positive and negative charges (Figure S1) [42,44]. In the case of ion exchange immobilizations, the enzyme–support linking will be beneficial at a pH different from the pI, as is the case for the lipases TLL, RML and Lecitase^®^ that, having more negative residues, achieved better immobilization yields using anion exchange supports [9,45] (Figure S1).

#### 3.1.1. Synthesis of Ethyl Esters of Fatty Acids Using Lipase Derivatives

The activity of the derivatives was evaluated according to their ability to catalyze the transesterification of fatty acids with ethyl alcohol to obtain ethyl esters of fatty acids (EE). In the Table 2 the results are shown in terms of %EE for the lipase derivatives obtained.

As a general trend, cation exchange immobilizations did not produce active derivatives. This may be because, during immobilizations performed at an acidic pH, a general pattern was observed, since the studied lipases gradually formed a precipitate accompanied by a more significant decrease in activity than in other conditions; see Table S1 (see Supplementary Material) [46,47]. Other effect to consider is the that ligands of the cation exchange supports: groups such as sulfonates or phosphonates have been reported to bind to the catalytic serine of hydrolase enzymes covalently, mimicking the second tetrahedral intermediate that occurs during the catalytic cycle, thus inactivating the enzyme [48]. Hence, it is reasonable to consider that the sulfonyl groups present in the support could also exert a comparable role within the transesterification reaction. This phenomenon, coupled with the reduced stability of lipases at acidic pHs, can synergistically contribute to the production of derivatives with lower EE yields (EE < 5%, Table 2) [15,48].

The activity of derivatives with hydrophobic interactions are those that produced the highest %EE (Lewatit^®^ VPOC1600- TLL 86.2%). On the other hand, the derivatives obtained via anion exchange also presented a %EE comparable to the commercial lipase derivatives Novozyme^®^ 435 (52.7%) and Lipozyme^®^ TL IM (71.3%). It is important to consider that in the industry, alcohol is typically added gradually during transesterification to prevent enzyme inactivation. [7]. The conditions used here are expected to be more challenging to biocatalysts as all alcohol is added at the onset of the reaction, which would explain the relatively low values for Novozyme^®^ 435 (52.7%) with regard to what has been previously reported [6,8,49].

Focusing on the hydrophobic supports that are the most common for lipases [44,49,50,51], the lipase derivatives immobilized on Lewatit^®^ VP OC 1600 were compared with Novozyme^®^ 435 (which is based on Lewatit^®^ VP OC 1600) and Lipozyme^®^ TL IM (based on silica) (Table 2 and Figure S2) [49,51,52] as commercial references. The best results in %EE were obtained with TLL, both the commercial reference and the one obtained here, with a higher percentage (71.3% and 86.0%, respectively) compared to Novozyme^®^ 435 (52.7%). Regarding the anion exchange derivatives, it should be noted that the PEI-Lecitase^®^ derivative also had comparable results (68.4%) to the reference. However, this was the only combination with which said enzyme achieved this performance, which shows that Lecitase^®^ is less versatile than TLL, which, in supports such as Lewatit^®^ VP OC 1600, Q-Sepharose and Lewatit^®^ MP800 (+), showed higher yields. Considering that the latter commercial supports are easily accessible, derivatives based on TLL sound promising for immediate application to biodiesel production at an industrial scale [7,21,50].

After selecting TLL as the most versatile lipase in this study, and upon comparing the EE yields of the respective derivatives (Table 2 and Figure S3), it is evident that, overall, those derived from anion exchange supports exhibited the most favorable outcomes (51% to 70%). This observation holds true with the exception of the derivative originating from PEI-agarose, with its yields being akin to those achieved using the commercial biocatalysts. This suggests that quaternary amino-type cationic groups facilitate the stabilization of a highly active form of TLL once immobilized, whereas secondary amino groups (such as those found in PEI-agarose) achieve this to a lesser extent [53]. On the other hand, structurally, the ligand in Q-Sepharose^®^ has methyl and methylene groups that can make the local environment more hydrophobic, which together with quaternary ammonium could simulate the activating effect that interfaces or surfactants have on lipases (e.g., CTAB) [25,41,43].

While the derivatives are intended for use in the synthesis of the alkyl esters of fatty acids (EE), saline enzyme–support bonds present in derivatives as Q-Sepharose^®^ are ideal when compared to the hydrophobic ones, because they may grant the derivative the advantage of being less susceptible to presenting the phenomena of enzyme leakage or poisoning via the adsorption of the predominantly hydrophobic species present during transesterification. In addition, the matrix selection was also one parameter to consider [42,53]. In this sense, hydrophilic agarose-based supports tend to present less adhesion effects on the oil components used on the surface of the derivative. Also, agarose-based supports such as Sepharose^®^ are obtained from renewable resources and biodegradable. Thus, this matrix emerges as a remarkable alternative with restricted application in the realm of biocatalyst design for EE synthesis. Our prior utilization of glyoxyl-agarose stands as one of the scarce instances of this [34]. Taking into consideration that one of the objectives of this study is to utilize additives like surfactants, which have demonstrated the ability to modulate lipase behavior, and to observe how these effects are manifested in the obtained derivatives, opting for a hydrophobic support is not the most suitable choice. This is because, in these cases, surfactants tend to diminish the quantity of enzyme that can be immobilized onto such supports, leading to derivatives with reduced activity [42]. Another reason to avoid hydrophobic supports is to prevent the immobilized lipase aggregation induced by short-chain alcohols [19]. Thus, the combination of Q-Sepharose^®^, a hydrophilic agarose-based support, with TLL, a versatile enzyme, holds promising potential, making it the central focus for studying the effects of additives on both of these, as will be seen below.

### 3.2. Effects of the Ionic Additives CTAB, SDS, PEI and CMC on the Hydrolytic Activity of TLL

These kind of additives have shown a capacity for tuning lipase properties as demonstrated previously for immobilized TLL in hydrolysis and for CALB in fatty acid ester synthesis [25,54,55]. Furthermore, they are readily available on the commercial market and widely utilized in both research and industrial applications [56,57]. Studying the impact of these additives on free TLL will also assist in defining the optimal conditions for immobilizing the enzyme (Section 3.3) onto the selected support, ultimately yielding derivatives with improved properties.

**Figure 1:**
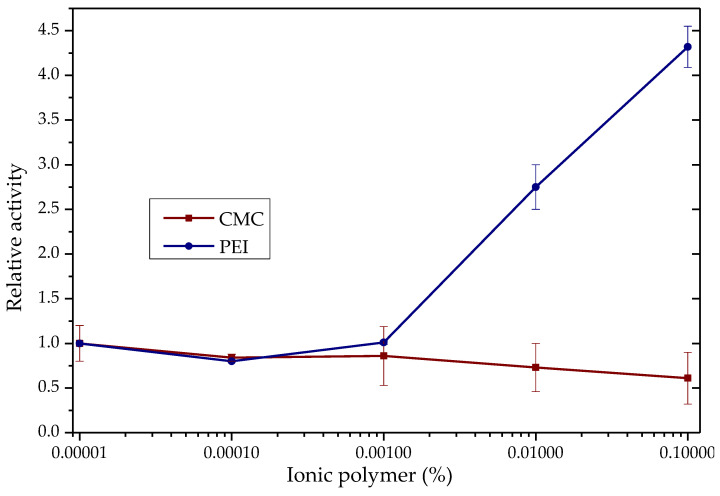
TLL p-NPB hydrolytic activity in the presence of CMC (red) and PEI (blue). Enzyme activity in the absence of polymer = 1 (0.037 IU). Measurements at pH 7.0 and 25.0 °C.

TLL shows changes in its activity in the hydrolysis of *para*-nitrophenyl butyrate (*p*-NPB) in the presence of ionic polymers such as poly(ethylenimine) (PEI) and carboxy-methylcellulose (CMC), Figure 1. With PEI, enzyme activity increased 4.3 times, while with CMC, a 39% decrease in activity was observed, each to 0.1% p/v.

**Figure 2:**
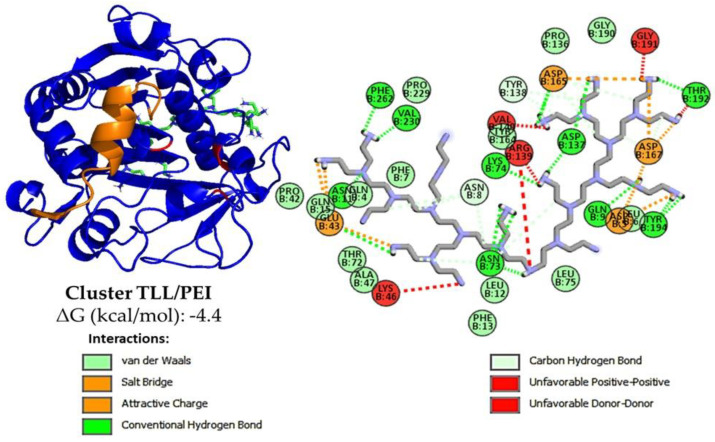
Representative cluster using AutodockVina of TLL in open conformation PDB: 6XOK (in blue). Oligomers were used as these are a representation of the polymer but with a reduced freedom degree, otherwise calculations will not converge. Here it is observed that PEI oligomers with *n* = 29 (represented in green) prefer to dock on regions with mainly enzymatic surfaces and not on regions near the active site (in red) or the domain of the lid (orange), this may be due to the large size of the oligomer chosen or the number of charges it has. Also, using Discovery Studio, the interactions are here represented in 2D: van der Waals (green light), salt bridge and attractive charge (orange), carbon hydrogen bond (blue light) and unfavorable interactions (red).

At pH 7.0 of the activity determinations, the TLL surface is negatively charged (between −5 and −12 of the net charge) [58], so the most intense additive–enzyme interactions would be, in the case of the polymers used, with the PEI, which is a polycation [38,58]. With the results, a molecular docking analysis was performed using an open conformation TLL structure (PDB:6XOK) and a representative oligomer structure of the CMC and PEI ionic polymers (Figure 2 and Figure S4). It can be deduced that the electrostatic interaction PEI-TLL could be preventing the formation of protein aggregates through their hydrophobic pockets, mainly by modifying lipase–lipase interactions and allowing the active site of the individual enzymatic units to be more available for binding to the substrate, that is, to present a greater activity [38]. Similar to that observed in Section 3.1.1, groups with positive charge improve enzyme activity, whereas groups with negative charge do not, which is a reasonable explanation of the mild promoter effect of these additives on the hydrolytic activity of lipase; it is not directly related to the active site domain (Figure 1) [22,23,59]. The observed increase with the CMC may be due to the opposite case of this phenomenon: the repulsion caused by similar charges between CMC and TLL could be favoring the formation of lipase–lipase aggregates through their hydrophobic pockets, thus decreasing the total activity of the enzyme [58,60]. On the other hand, although the energy, according to the results in Figure 2, proposes that the cluster with CMC is more stable than that of PEI, this is most likely due to the constraints of the docking process, wherein the employed force field fails to consider ionic interactions [61].

**Figure 3:**
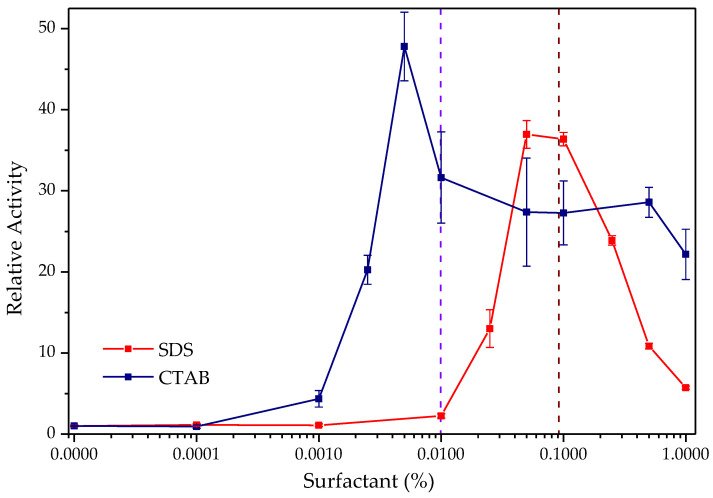
Hydrolytic activity of TLL against *p*-NPB in the presence of CTAB (blue) and SDS (red). Relative enzyme activity in the absence of surfactant = 1 (0.054 IU for SDS and 0.107 IU for CTAB). The dotted brown and light blue lines represent the approximate value of critical micelle concentration for the SDS and CTAB, respectively, in SBF and 25.0 °C (3 mM).

As previously reported, TLL experiments display a drastic increase in hydrolytic activity in the presence of ionic surfactants, as seen in Figure 3 [25,29]. The highest activation was obtained with the cationic surfactant CTAB, approximately 48 times that of the enzyme’s activity without the surfactant and at a relatively low concentration of the surfactant (0.005% (*w*/*v*)). The anionic surfactant SDS also promoted considerable activity, 37 times higher than the enzyme without the surfactant, but at higher concentrations (0.05–0.1%), compared with CTAB.

In both cases, a similar initial growth behavior is observed in catalytic activity, followed by a decrease to higher concentrations of surfactants, although consistently superior to control. Similar hyperactivation observations have been obtained for this lipase in previous studies with concentrations of CTAB (0.005%) [25] and SDS (0.1% ≈ 3 mM) [29,62], in addition to the behavior of its activity profile in broad concentration ranges of these surfactants. This is related to the relative maximum point of activation reached by concentrations close to the critical micellar concentration value of each of the surfactants in the phosphate-buffered solution (SBF) that was used for the determinations [63,64]. This would seem to indicate that the observed activation phenomenon is due to the adsorption of the micelle on lipase in its open form that appears when surfactant micelles are formed (interfacial activation). However, Mogensen et al. demonstrated that it is the interaction of individual surfactant molecules with critical points of the enzymatic surface that is responsible for this phenomenon; no evidence of the formation of micelles is found or, including premicellar aggregates, at the surfactant concentrations at which hyperactivation occurs [29].

**Figure 4:**
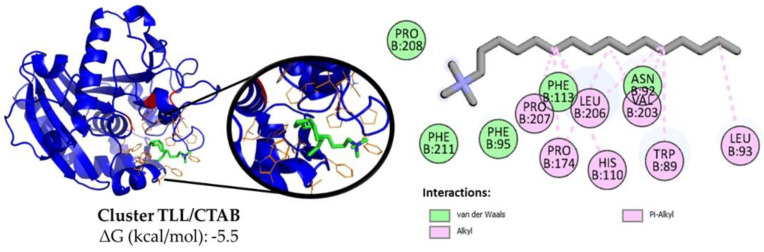
Representative cluster using AutodockVina between open TLL (blue) and CTAB (green/red/orange). The catalytic triad (red) and the residues closest to the detergent (orange) are highlighted. Also, using Discovery Studio the interactions in 2D are here represented: van der Waals (green light) and alkyl and Pi-Alkyl (pink).

In contrast to the docking outcomes observed for ionic polymers, the interaction between CTAB (ΔG = −5.5 kcal/mol) and SDS (ΔG = −5.2 kcal/mol) with the active site of TLL yielded negative free energy values (ΔG < 0) (Supplementary Figures S5 and S6). This suggests a pronounced spontaneous tendency for the positioning of these surfactants within the enzyme’s hydrophobic pocket, aligning well with the findings reported in the existing literature [25,27,28,29] and in accordance with experimentally obtained activity results. The main location of its carbon chain appears to be next to the hydrophobic residues such as Trp, Phe, Leu and Ile that surround the catalytic triad of the enzyme (Figure 4 and Figure S5) [59,65,66,67], allowing it to establish strong van der Waals interactions, while its unique ionic end is exposed to the medium. In addition, the TLL-surfactant clusters obtained with the substrate *p*-NPB were evaluated using a receptor (Figure S7), which revealed that the presence of the surfactant in the active site domain does not interfere with the affinity it has to the ester for both cases (ΔG = −5.6 kcal/mol).

On the other hand, it is observed that, although both surfactants have the ability to significantly increase hydrolytic activity, the activation presented using CTAB was higher (48 times CTAB/37 times SDS); this could be due to an acceleration of the reaction by the formation of a cationic complex between the product of *p*-NPB hydrolysis and a molecule of CTAB (Le Châtelier principle) [68,69]. In addition, beyond the critical micelle concentration, there is a decrease in hydrolytic activity allegedly attributed to the fact that once micelles are formed, they slightly reduce the effective concentration of the substrate because a portion of hydrophobic characteristic of the substrate may be encapsulated in the micelle [70].

### 3.3. TLL Immobilization on Q-Sepharose^®^ Supports Using Ionic Surfactants

As mentioned in Section 3.2, lipases have a conformational balance between an inactive closed and active open form, which is also evidenced in slightly immobilized lipases [25,26,66]. Although the closed form is the most stable, the active form can be stabilized in the presence of ionic surfactants. In addition, it has been shown that the use of this type of ionic surfactant not only activates the enzyme [25,66,70], because the hydrophobic rest of the surfactant can remain adsorbed in the active site, but the charged rest is also adsorbed in an ionic charge exchanger opposite to the surfactant. Thus, surfactants may connect the active lipase form with the ionic support [25]. Taking advantage of the results discussed in Section 3.1 and Section 3.2, that the polymer’s effect was diminished compared to the surfactants, here the impact of the ionic surfactants on the immobilization of TLL onto Q-Sepharose^®^ was investigated due to its potential to yield positive effects in the resulting derivatives.

#### 3.3.1. Effect of Ionic Surfactants on the Immobilization Process

**Figure 5:**
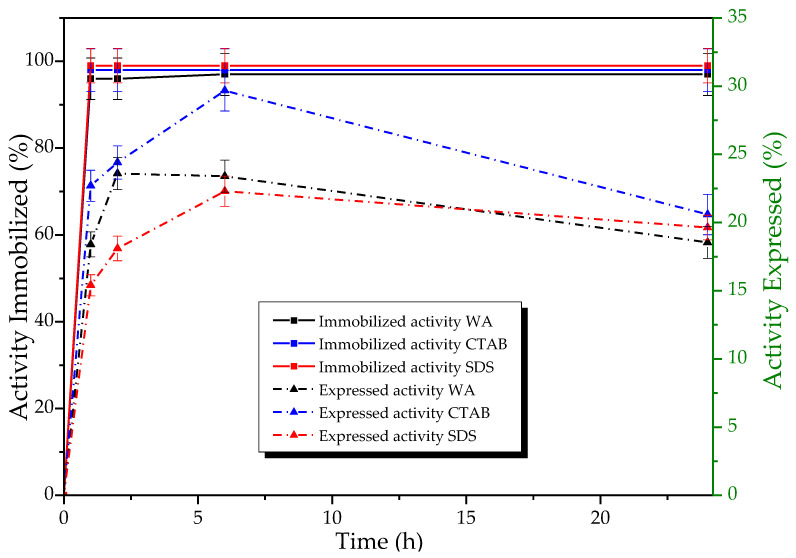
Percentage of immobilized hydrolytic activity (p-NFB) (**Left Panel** continuous line of squares) and expressed activity (**Right Panel** dotted line of triangles) of TLL derivatives in Q-Sepharose^®^ concerning the immobilization target, in the absence (WA), and presence of additives (CTAB, 0.005%; SDS, 0.1%; PEI, 0.1%), pH 7.0 at 28 °C. A total of 1.86 mg of protein per g of support was offered, and the activity offered for each case was: Q/WA: 2.9 ± 0.2 UI, Q/CTAB: 3.8 ± 0.1 UI, Q/SDS: 1.89 ± 0.04 UI. Expressed activity was defined as the percentage difference between the activity of the derivative (Xed) and the activity of the supernatant (Xsb) divided by the activity of the immobilization control (Xcs) (enzyme solution mixed with non-activated agarose) and the activity of the derivative (Xed) (100 (Xed − Xsb)/Xcs) [34].

This exploration is particularly relevant because both the Q-Sepharose^®^ support and the additives have demonstrated the capability to enhance the activity. Figure 5 shows the results obtained in monitoring the immobilization process in the absence (WA) and the presence of the different ionic surfactants tested with the free enzyme.

High percentages of immobilization (84–99%) were obtained for TLL in the anion exchange support for all immobilization conditions without significant differences. In contrast, the results of the activity expressed vary during immobilization (Figure 5), tending to increase as the immobilization time does. The period of 24 h was chosen as the expressed activity was almost maintained and as the longer the immobilization time, the higher the derivative’s stability against deactivating agents [71], which is desirable in the challenging conditions of the production of EE.

Once the derivatives had been washed, the esterase activity of each derivative in the absence of surfactant was determined, which was generally too low to make direct comparisons between them. Therefore, it was necessary to add ionic additives to make said comparison. However, it is noteworthy that, in contrast, the TLL derivative obtained using SDS in Q-Sepharose^®^ showcased remarkable activity, even in the absence of modifiers (1.02 UI). This value stands notably higher—ranging between 11 to 102 times—than the activity levels observed in other prepared derivatives (ranging from 0.010 to 0.09 UI). Impressively, this activity is also 93 times greater than that exhibited by the free enzyme during its corresponding 24 h immobilization target (0.011 IU) (Table 3).

**Figure 6:**
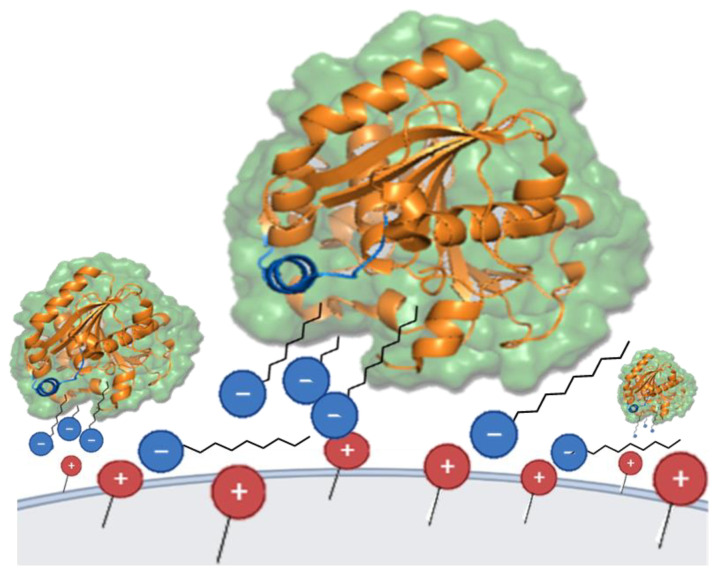
Immobilization representation of TLL on Q-Sepharose^®^ support in its open form through surfactant molecules (SDS, blue). The illustrated phenomenon scheme draws parallels to ion-pair chromatography [74], although it is characterized by the distinction that, in this instance, ion-pairs form between the stationary phase (embodied by the support) and the surfactant or surfactant–protein complexes. In turn, this interaction would hypothetically result in the apparent hydrophobization of the support, thus mimicking an oil/water interface that could potentially activate lipases, such as TLL.

The remarkably elevated esterase activity observed in Q-TLL/SDS suggests that, under these specific conditions, a substantial quantity of the enzyme was successfully stabilized upon immobilization in its hyperactivated state. This heightened activity persists both after immobilization and even in the absence of the additive SDS. This phenomenon, referred to verbatim as “bio-imprinting” by certain authors [72,73], appears to be inapplicable to derivatives obtained here in the absence of SDS. Similar observations were obtained for TLL immobilized on sulfopropyl- Sepharose^®^ (SP), a cation exchanger, in the presence of CTAB at relatively high concentrations (0.3%) [25]. Although the immobilization mechanism is not yet determined on a molecular scale, this may involve an enzyme–surfactant–support interaction, in which the surfactant forms a kind of cluster bridge due to its hydrophobic end interacting with the active site of the enzyme and its ionic end adsorbing strongly with to the surface of the carrier with the opposite charge (Figure 6) [25].

**Figure 7:**
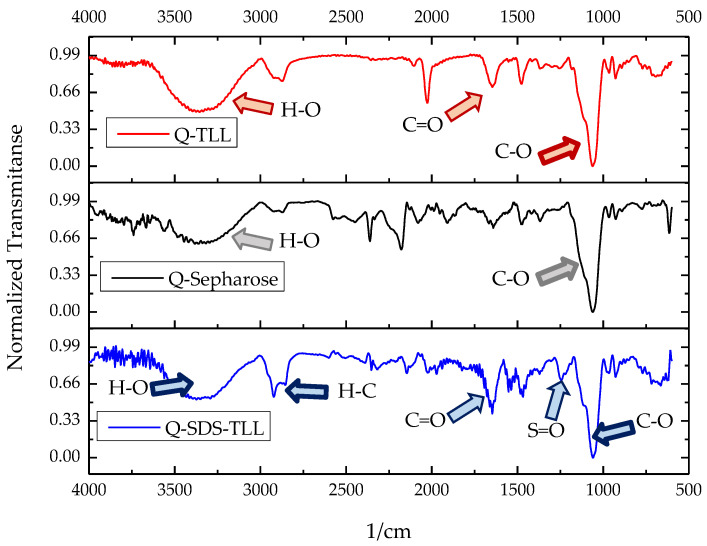
Comparative FTIR-ATR normalized transmittance spectra of derivative Q-Sepharose^®^-TLL (Q-TLL, red), Q.Sepharose^®^ (gray) and derivative Q-Sepharose^®^-SDS- (Q-SDS-TLL, blue). For TLL derivatives, the carbonyl band (C=O) from the peptide bonds of the TLL enzyme is well known. The spectrum of the derivative Q-SDS-TLL is the only one that appears as evidence of the presence of the surfactant SDS: stretching S=O (1300 cm^−1^) and C-H (2900 cm^−1^).

To add evidence to the aforementioned enzyme–surfactant–support interaction, TLL derivatives immobilized on Q-Sepharose^®^ in the presence of SDS were characterized using FTIR (Figure 7). The characteristic bands of the support in the infrared spectrum can be observed as they are the stretching O-H in 3300 cm^−1^ and C-O in 1100 cm^−1^. Comparing the spectrum of the Q-TLL and Q-SDS-TLL derivatives with the support without an enzyme, it is observed that for both cases, the presence of enzyme is reflected in an increase in the band of 1620 cm^−1^ due to the presence of the peptide bonds of TLL [34]. For its part in the spectrum of the derivative Q-SDS-TLL, the bands can be seen in 2900 cm^−1^, characteristic of the alkanes, and 1300 cm^−1^ of the stretch S=O, thus demonstrating the appearance of SDS as a constituent of the lipase derivative (Figure 7). As the derivatives were washed concomitantly and filtered before this characterization, this demonstrated that SDS is indeed bound to the support, probably through ionic interactions, or to the enzyme via hydrophobic interactions.

**Figure 8:**
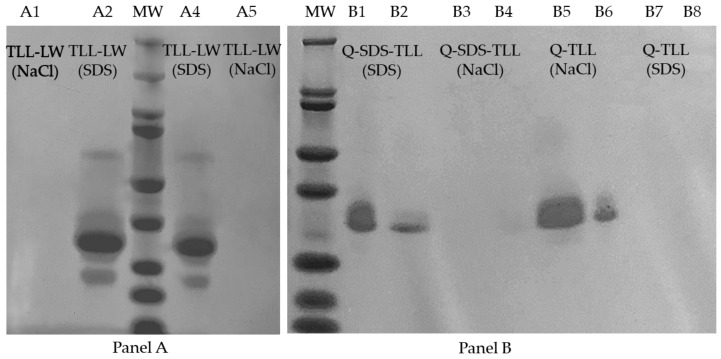
Images of SDS-PAGE gels of derivative supernatants, in order from left to right. (**Panel A**), Lane A1 and A5: TLL-LW using NaCl, Lane A2 and A4: TLL-LW using SDS, and MW: BioRad molecular weight marker. (**Panel B**), MW: BioRad molecular weight marker, Lane B1 and B2: Q-SDS-TLL using SDS, Lane B3 and B4: Q-SDS-TLL using NaCl, Lane B5 and B6: Q-TLL using NaCl, and Lane B7 and B8: Q-TLL using SDS.

One way to corroborate which type of interactions are predominant in the enzyme–support derivative is to find the enzyme desorption conditions from the support [75,76]. Q-TLL and Q-SDS-TLL derivatives, along with typical hydrophobic derivatives (TLL-LW), were subjected to as harsh desorption conditions as those used during the preparation of samples for denaturing electrophoresis, such as high temperature (Section 2.6) and including 4% (*w*/*w*) SDS or 1 M NaCl, the first to undo hydrophobic interactions and the second for the ionic type. The obtained supernatants were injected into SDS-PAGE gels (Figure 8), revealing whether the enzyme desorbs or remain bonded under the condition employed.

The results of SDS-PAGE show an intense band at 33 kDa, similar to the molecular weight reported in the literature for TLL [77]. In the case of the hydrophobically interacting derivative, TLL-LW, enzyme desorption can be accomplished solely with the use of SDS. However, the presence of NaCl fails to induce enzyme desorption from LW (Lewatit^®^ VPOC1600). Conversely, for the ionic derivative Q-TLL, an opposite pattern of behavior is observed [75,76]. Of particular interest, the Q-SDS-TLL derivative, in contrast to the behavior observed in Q-TLL, exhibits a notably pronounced desorption when subjected to SDS compared to NaCl. This distinctive response is attributed to the nature of the interactions at play, highlighting that within the Q-SDS-TLL derivative, the enzyme primarily forms hydrophobic interactions with the support due to the presence of immobilized SDS (Figure 6).

In addition, in terms of esterase activity, the effect of different concentrations of additives on Q-TLL derivatives was also evaluated. In general, they responded similarly to the free enzymes. Table 4 summarizes the most representative results of this study and in Figures S8 and S9 (see Supplementary Material), the respective activity curves for each of the seven derivatives are presented.

**Figure 9:**
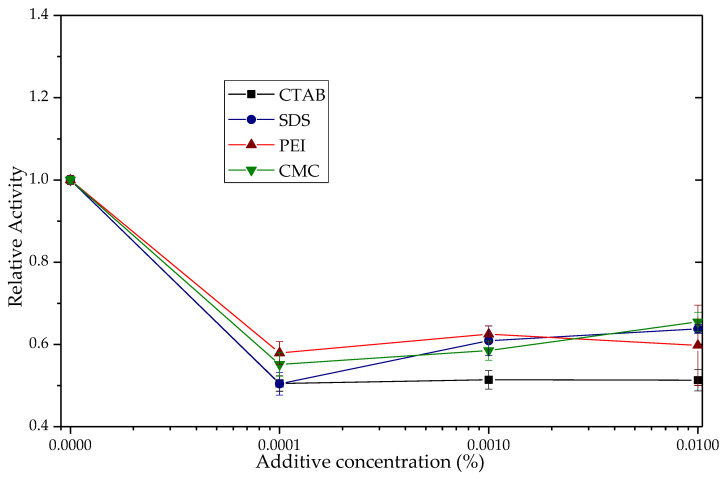
Esterase activity of the TLL derivative Q-Sepharose^®^/SDS in the presence of CTAB, SDS, PEI and CMC. The relative activity of the derivative in the absence of additives was 1 (1.02 UI). The point-to-point junction serves solely for the convenient visualization of the graph.

As mentioned in Table 3, Section 3.3.1, the derivative Q-Sepharose^®^/SDS was the one with the highest activity in the absence of additives, evidencing bio-imprinting. That may explain why for this derivative the esterase activity results, shown in Table 4 imply a slight activation or even a lower activity when adding the additives (Figure 9). It was only through the addition of a relatively elevated quantity of CTAB (0.1%) that a marginal rise in activity (20%) was discernible. This could be attributed to the hyperactivation of a small fraction of the enzyme population, which was likely immobilized in its closed conformation, possibly via ionic interactions with the support. One of the most remarkable benefits of the Q-TLL-SDS derivative is its ability to avoid the necessity for surfactants or other additives to activate the immobilized enzyme, a crucial factor for potential hydrolysis applications. Furthermore, the final reaction products would no longer require separation from this type of additive, as they would reside within a different phase. Similarly, the utilization of this derivative in transesterification would enable something that cannot be achieved with other derivatives, which is the attainment the activating effect of SDS without the need to add it to the reaction medium. Implementing such an addition would result in the formation of emulsions that would complicate the purification process of biodiesel [78,79].

#### 3.3.2. Application of Q-TLL Derivatives in Ethyl Ester Production

**Figure 10:**
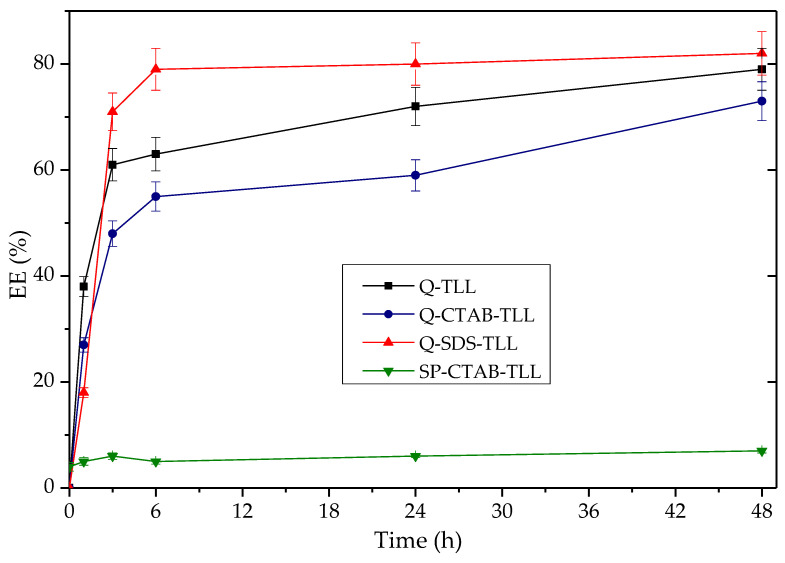
Time-course of the production of %EE from palm olein as a function of time taken for derivatives 20 mg TLL/gram support. Reaction at 37 °C at 1700 rpm using 40 mg by mass of derivative (% 6 p/p against the amount of oil). Molar ratio 3.1: 1 EtOH: palm olein. For all EE synthesis experiments, no additional surfactant was added other than that used to obtain the derivative previously.

Regarding % of EE obtained as a function of time using the derivatives Q-TLL, Q-CTAB-TLL and Q-SDS-TLL (Figure 10), the maximum production is around 70–80% in all cases. It should be noted that for the derivative Q obtained with SDS, in the process of immobilization (Q-SDS), the highest %EE (~80%) was obtained at 6 h, in contrast to the other two derivatives that needed 48 h of reaction to reach %EE~70%. This is evidence of this type of derivative’s potential, consistent with the high hydrolytic activity found in Section 3.3.1 in the absence of surfactants in the reaction medium. This is, in turn, another indication of the bio-imprinting phenomenon for Q-SDS-TLL that is maintained even in the non-conventional medium of the transesterification reaction [25]. It is worth noting that the SP-CTAB-TLL derivative previously characterized as bio-imprinted [25] did not exhibit significant EE production (Figure 10). This might be attributed to CTAB potentially obstructing the ingress of triacylglyceride (TG) into the active site, primarily due to its aliphatic chain possessing a greater number of carbons (16), in contrast to the chain length of SDS (12) [80]. Consequently, this could explain why both derivatives demonstrate comparable behaviors with diminutive substrates like *p*-NPB [25], while showing disparity with larger substrates such as the triacylglycerides present in the transesterification reaction.

**Figure 11:**
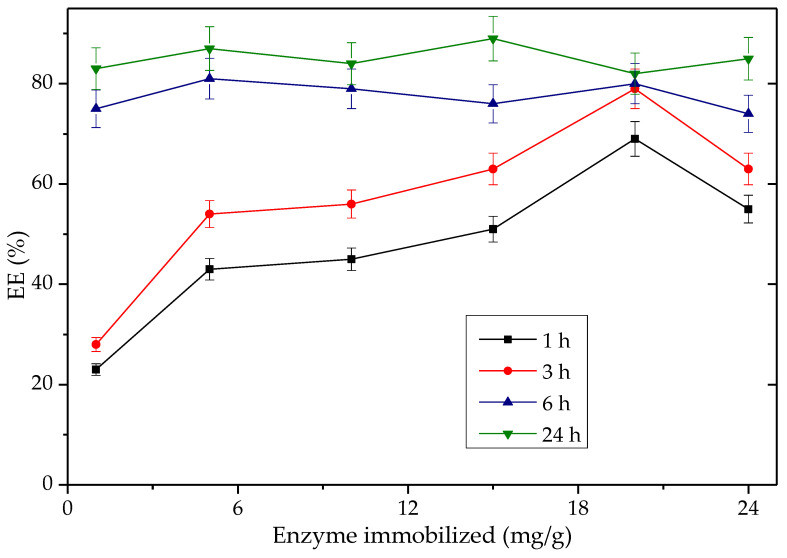
Time-course tracking of palm %EE production over time for Q-SDS-TLL derivatives with the amount of enzymes immobilized ranging from 1 mg TLL/g support to 25 mg TLL/g support. Reaction conducted at 37 °C and 1700 rpm, utilizing 40 mg of the derivative in the reaction vessel. For all EE synthesis experiments, no additional surfactant was added other than that used to obtain the derivative previously.

Building on its impressive performance, the Q-SDS-TLL derivative was chosen for subsequent studies, including variations in enzyme concentration, reaction mixture and exploring its reusability in transesterification. These investigations aim to enhance the biocatalyst’s efficiency, with the goal of bolstering its potential for future large-scale production. Figure 11 and Figure S10 (see Supplementary Material) show the production of EE with derivatives obtained by varying the protein load between 1 mg TLL/g support and 25 mg TLL/g support. Taking a reaction time of 3 h, as expected, they all show proportionality between the amount of EE% synthesized and the concentration of immobilized enzyme in the derivative, except for the 25 mg/g derivative. The latter can be explained due to increasing the amount of enzyme above a specific maximum value (~20 mg/g); aggregation effects at the surface level of the support could be promoted, leading to less enzymatic activity and also minimizing the effectiveness of bio-imprinting by the effect of such an interaction between proteins. The lower specific activity (%EE/mg immobilized protein on the support) for derivatives with a greater amount of immobilized protein per gram of support could be also related, with a greater difficulty for the substrate to saturate all available active sites by the effect of the mass transfer phenomena [81,82]. It was important that the specific activity of the derivative of 1 mg protein/g became up to 25 times higher compared to the rest once the 6 h reaction threshold was exceeded. It is worth noting that the derivatives Q-TLL and Q-CTAB-TLL had low percentages of EE (~31%) when tested using enzyme charges below 20 mg/g, see Table S2 (Supplementary Materials), which reinforce the choice of Q-SDS-TLL out of the Q-Sepharose^®^ derivative types. Considering the results of Figure 11, the enzymatic load 1 mg/g for Q-SDS-TLL was chosen, given its consistent high specific activity and the low amount of enzyme required.

**Figure 12:**
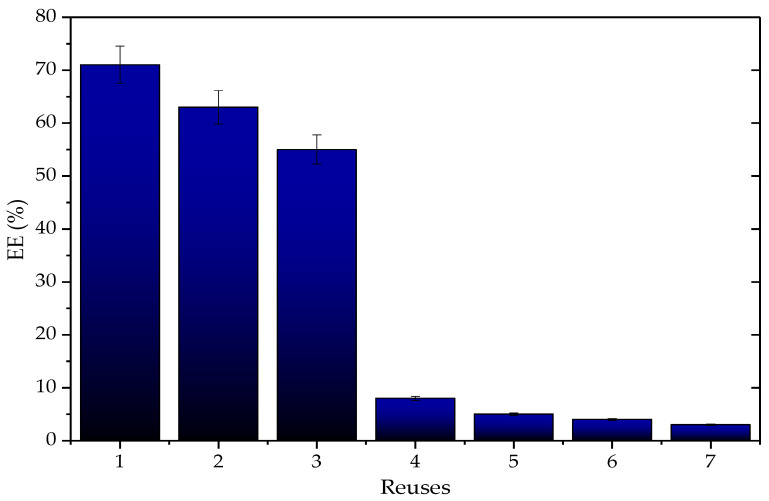
Reuses of the derivative type Q-SDS-TLL regarding %EE produced; each cycle represents one use of the biocatalyst subjected to reaction for 3 h. Reaction at 37 °C at 1700 rpm using 40 mg by mass of derivative with 1 mg/g charge. For all EE synthesis experiments, no additional surfactant was added other than that used to obtain the derivative previously.

**Figure 13:**
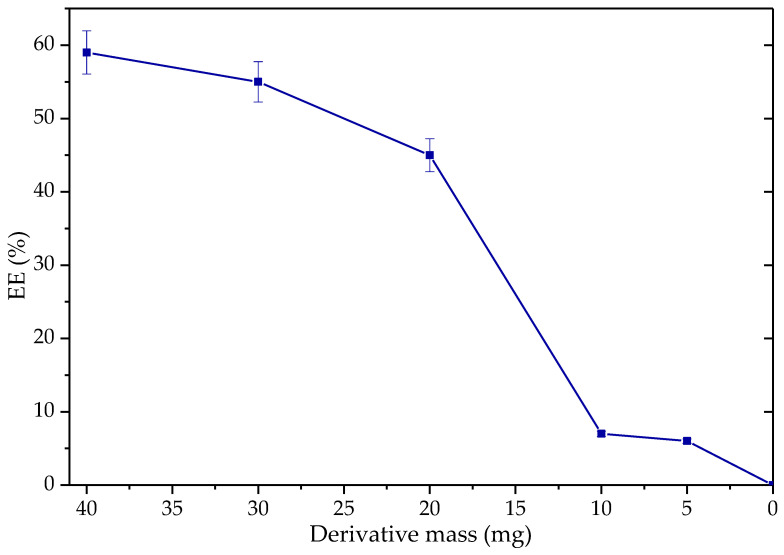
Production of ethyl esters as a function of derivative mass (Q-SDS-TLL with 1 mg TLL/g support). Reaction at 37 °C at 1700 rpm, molar ratio 3.1: 1 EtOH: palm olein (from 0.8–6% *w*/*w*) for 6 h. For all EE synthesis experiments, no additional surfactant was added other than that used to obtain the derivative previously.

As part of the evaluation of the reusability of the Q-SDS-TLL derivative, assays of EE synthesis were conducted. Figure 12 shows the percentage decrease in synthesized EE in each cycle. According to these results, up to the third cycle the EE yield losses did not exceed 20%, however, after the fourth use, there was a considerable decrease. No evidence of desorption of the enzyme in the derivatives was obtained (the amount of the protein content in the derivative was maintained, and the hydrolytic activity per gram as well), unlike in previous work with the derivative TLL-LW [9]. This decrease is mainly attributed to the visually observed loss of the derivative mass between each cycle as a result of the filtration and washing operations that had to be carried out. These losses could not be quantified due to the low amount of biocatalyst used under the reaction conditions (40 mg). It is expected that when larger reaction scales are used, or when using a continuous reactor, the proportion of derivative mass lost due to reuse will be proportionally less than the one observed here [83]. These results led us to study the effect of the amount of derivative on the EE yield (Figure 13).

As expected, increasing the amount of derivative in the reaction mixture increases its speed almost linearly in a period of 6 h (Figure 13). In more extended periods (24 h), EE production is not significantly affected by reducing the mass amount between 40 and 20 mg of derivative. However, by reducing the quantity below 20 mg, which is 3% *w*/*w* for oil, the EE generated decreased abruptly to below 10%EE. This is because, during the experiment, it was observed that below these quantities, the dispersion of heterogeneous catalysts within the reaction mixture was higher, causing the particles to be more exposed to the ethanolic phase, inhibiting the process. Hence, it appears that the decrease in EE yield during the reuse experiments may indeed be a result of a decrease in the derivative mass in the reaction vessel.

### 3.4. Comparison of EE Production of the Derivative Q-SDS-TLL Versus Lipozyme^®^ TL IM

This section shows the behavior of Q-SDS-TLL and a commercial biocatalyst based on TLL against oils that could not be used in industrial biodiesel production unless pretreated. Table 5 shows that the behavior of Q-SDS-TLL (with just 1 mg protein immobilized/g) is similar to using crude palm oil and greater in used palm oil compared to that of the commercial biocatalyst (42 mg immobilized protein/g), but with much lower enzyme expenditure. The %EE is lower in unrefined oil presumably because in the unrefined oil there may be phospholipids that can inhibit lipase activity [84].

The use of lower-quality oils for biodiesel production is one of the aspects that has been mentioned as critical in the sustainability of this fuel in the future, since this raw material constitutes the highest cost of production [10,85]. The need to use high-quality oils as operational requirements for homogeneous acid or basic catalysts used in the global biodiesel industry presents ethical dilemmas such as those related to using food in fuel production [10], something that would be mitigated by biocatalysts based on promising derivatives such as Q-SDS-TLL. The design of novel biocatalysts for EE with agarose-based supports containing a quaternary amino group and SDS as an immobilization additive form an effective strategy to improve biocatalyst EE yields. This was recently tested when TLL was immobilized on a modified glyoxyl-agarose support that, in addition to aldehyde groups, had quaternary amino groups (GxGT). The same improvement in %EE was obtained by adding SDS as an immobilization additive: 34.6% for GxGT-TLL to 64.2% for GxGT-SDS-TLL (Table S3 (see Supplementary Material)). These novel supports and their derivatives have, additionally, the advantage of promoting covalent immobilization which is expected to improve biocatalysts’ performance, mainly in their stability under even harsher reaction conditions, something that will be exploited in future works.

## 4. Conclusions

Among the numerous evaluated derivatives for ethyl ester (EE) production, *Thermomyces lanuginosus* (TLL) lipase showed remarkable versatility. It successfully immobilized on both ionic and hydrophobic supports, delivering EE yields comparable to commercial alternatives with reduced enzyme expenditure. Incorporating ionic additives enhanced TLL’s hydrolytic activity, while molecular docking indicated that surfactants stabilize the open lipase conformation. Immobilizing TLL on Q-Sepharose^®^ using surfactants led to varied derivative hydrolytic activity. Of the combinations evaluated, it was highlighted that in the case using SDS, a biodegradable and commercially available additive [57], in the immobilization process of TLL on Q (Q-SDS-TLL) this combination exhibited the highest activity (1.02 UI), probably by retaining its open conformation even without adding extra additives, potentially indicative of enzymatic “bio-imprinting”. Q-SDS-TLL also excelled at minimal enzyme consumption (1 mg/g of support), displaying a good performance in EE synthesis without using solvents and without the requirement more additives than the one already bound to the derivative constituents, achieving comparable results than those obtained using commercial derivatives such as Novozyme^®^ 435, which use 82 times more enzyme. This was observed even using used oil (79.3% compared with 77.8%). Leveraging continuous bio-reactors might overcome reuse limitations, elevating the competitiveness of biocatalysts. Future work could optimize the conditions for target EE yields (>96.5%). When comparing Q-SDS-TLL’s EE yields with those obtained from refined, unrefined, or used oils using other catalysts, it becomes evident that its advantages could eventually lead to cost reductions and a decrease in the environmental impact of current biodiesel production practices.

## Figures and Tables

**Table 1:** Immobilized activity yield (%) of the lipases TLL, RML, CALB and Lecitase^®^ on anionic, cationic and hydrophobic exchange supports, in 24 h of immobilization. In the matrix type, the type of interaction with the enzyme and the hydrophilic or hydrophobic characteristic of the matrix is detailed.

Support	Abbr.	Matrix	Enzyme	Immobilization pH	% Activity Immobilized ^a^
Sulfopropyl Sepharose^®^ (−)	SP	Crosslinked agarose Ligand: Sulfopropyl Type: Anionic, hydrophilic	TLL	3.5	87.9
			CALB	3.5	81.3
			RML	3.5	87.7
			Lecitase^®^	3.5	74.3
Q-Sepharose^®^ (+)	Q	Crosslinked agarose Ligand: Quaternary amine Type: Cationic, hydrophilic	TLL	8.0	85.9
			CALB	10.0	70.6
			Lecitase^®^	8.5	41.5
Dextran Sulfate agarose (−)	DexSO_4_	Crosslinked agarose Ligand: Sulfonic acid Type: Anionic, hydrophilic	TLL	3.5	85.9
			CALB	3.5	79.6
			RML	3.5	69.6
			Lecitase^®^	3.5	58.0
Octyl-Sepharose^®^ *	OC	Crosslinked agarose Ligand: Octyl groups Type: Hydrophobic, hydrophilic	TLL	7.0	45.0 [38]
			CALB	7.0	79.0 [39]
			Lecitase^®^	7.0	45.0 [38]
Polyethyleneimine—agarose (+)	PEI	Crosslinked agarose Ligand: Ethyleneimine Type: Cationic, hydrophilic	TLL	8.0	67.2
			CALB	9.5	25.2
			Lecitase^®^	8.5	94.1
Nekrolith^®^ (+)	NK	DVB/styrene tertiary amine groups [40] Type: Cationic, hydrophobic	TLL	8.5	74.5
			CALB	9.5	72.6
			RML	8.0	80.7
			Lecitase^®^	8.5	94.1
Lewatit^®^ VPOC1600	VPOC	DVB/Crosslinked polymethacrylate Ligand: None Type: Hydrophobic, Hydrophobic	TLL	8.0	87.7
			CALB	8.0	97.0
			RML	8.0	82.0
			Lecitase^®^	8.0	90.0
Lewatit^®^ MPSP112H	MPSP	DVB/Crosslinked polystyrene Ligand: Sulfonic acid Type: Anionic, hydrophobic	TLL	3.5	72.0
			CALB	3.5	30.0
			Lecitase^®^	7.0	80.0
Lewatit^®^ MP800	MP800	DVB/Crosslinked polystyrene Ligand: Quaternary amine Type: Cationic, hydrophobic	TLL	8.0	94.0
			CALB	9.5	27.0
			Lecitase^®^	8.5	82.0
Purolite^®^ ECR1604	PL^®^	Polystyrene Ligand: Quaternary amine Type: Cationic, hydrophobic	TLL	8.0	80.0
			CALB	9.5	26.0
			Lecitase^®^	8.5	83.2

DVB-Divinylbenzene. ^a^ The immobilization yield under defined immobilization conditions (Section 2.2) was calculated as 100% (1−XSI × XCS ^−1^), where XSI and XCS indicate the total activity content after the immobilization time in the supernatants that were in contact before the measurements with and without support, respectively [9]. In every enzyme immobilization 75 mg of protein per g support was offered. Values are the mean of three different experiments where the standard deviation was never >5% of the mean value.

**Table 2:** Yield (%) of ethyl esters fatty acids (EE) produced from each lipase derivative in 24 h of reaction, using 3.1:1 ethanol: oil, 37 °C, 1700 rpm [9].

Support	Enzyme	%EE	CI
Sulfopropyl Sepharose^®^ (−)	TLL	3.3	0.4
	CALB	4.4	0.6
	RML	2.9	0.4
	Lecitase^®^	3.6	0.5
Q-Sepharose (+)	TLL	70.1	5.6
	CALB	5.7	0.5
	Lecitase^®^	50.7	4.1
Dextran Sulfate agarose (−)	TLL	3.2	0.4
	CALB	4.4	0.6
	RML	3.4	0.4
	Lecitase^®^	3.7	0.5
Octyl-Sepharose^®^	CALB	32.4	2.6
Polyethyleneimine-agarose (+)	TLL	4.7	0.4
	CALB	35.8	2.9
	Lecitase^®^	68.4	5.5
Nekrolith^®^ (+)	TLL	51.3	4.1
	CALB	30.4	2.4
	RML	20.6	1.6
	Lecitase^®^	22.8	1.8
Lewatit^®^ VPOC1600	TLL	86.2	6.9
	CALB	47.4	3.8
	RML	70.7	5.7
	Lecitase^®^	11.4	0.9
Lewatit^®^ MPSP112H (−)	TLL	11.0	0.9
	CALB	9.0	0.7
	Lecitase^®^	10.0	0.8
Lewatit^®^ MP800 (+)	TLL	65.1	5.2
	CALB	27.2	2.2
	Lecitase^®^	55.0	4.4
Purolite^®^ ECR1604	TLL	54.2	4.3
	CALB	10.1	0.8
	Lecitase^®^	16.3	1.3
Novozyme^®^ 435		52.7	4.2
Lipozyme^®^ TL IM		71.3	5.7

Values are the mean of three different experiments where the standard deviation was never >5% of the mean value (Section 2.4). CI: confidence interval.

**Table 3:** Expressed activities of the derivatives obtained, measured in the absence of surfactants in the activity solutions. The activity relative to the immobilization target without additives (0.011 IU) is shown. Here comparisons were made using 0.06 mg of free or immobilized protein for measurement.

Derivative (Support/Additive during Immobilization)	Surfactant-Free Derivative Esterase Activity (UI)	Ratio between Expressed and Initial Activity
Q-Sepharose^®^/WA	0.09 ± 0.01	8.2
Q-Sepharose^®^/CTAB	0.048 ± 0.007	4.4
Q-Sepharose^®^/SDS	1.02 ± 0.09	92.7

**Table 4:** Representative results of the effect of surfactants and polymers on the esterase activity of different Q-TLL derivatives.

Derivative (Support/Condition of Immobilization)	Additive Added to the Esterase Reaction Medium (Concentration Yielding Maximum Activity.)	Maximum Activity Observed	
		Relative	(UI)
Q-Sepharose^®^/WA	Without additives	1	0.09± 0.01
	CTAB (0.1%)	33	2.98 ± 0.06
	SDS (0.01%)	14	1.24 ± 0.06
	PEI (0.1%)	1.8	0.161 ± 0.006
	CMC (0.1%)	1.4	0.13 ± 0.01
Q-Sepharose^®^/CTAB (0.005%)	Without additives	1	0.048 ± 0.007
	CTAB (0.1%)	61	2.92 ± 0.06
	SDS (0.01%)	30	1.43 ± 0.05
	PEI (0.1%)	2	0.095 ± 0.007
	CMC (0.1%)	2	0.086 ± 0.006
Q-Sepharose^®^/SDS (0.1%)	Without additives	1	1.02 ± 0.09
	CTAB (0.01%)	0.51	0.52 ± 0.1
	SDS (0.01%)	0.64	0.65 ± 0.01
	PEI (0.01%)	0.60	0.61 ± 0.03
	CMC (0.01%)	0.65	0.66 ± 0.02

**Table 5:** Comparison of EE production (%) of Q-SDS-TLL (1 mg/g) and Lipozyme^®^ derivatives using unrefined or used palm oil.

Unrefined Palm Oil			Used Palm Oil	
Time (h)	Q-SDS-TLL	Commercial Derivative Lipozyme^®^	Q-SDS-TLL	Commercial Derivativelipozyme^®^
1	28.5 ± 2.7	17.7 ± 0.3	28.1 ± 2.9	34.5 ± 3.2
6	45.9 ± 6.0	50.0 ± 5.8	62.6 ± 1.8	64.4 ± 1.7
24	67.5 ± 2.6	64.6 ± 4.0	79.3 ± 0.2	77.8 ± 0.8

## Data Availability

Not applicable.

## Supplementary Materials

The following supporting information can be downloaded at https://www.mdpi.com/article/10.3390/biotech12040067/s1, Figure S1: Representation of surface load density of lipases from *Thermomyces lanuginosus* (TLL), *Rhizomucor miehei* (RML), *Candida antarctic* B (CALB) and Lecitase^®^; the isoelectric points are 5.36, 4.92, 8.12 and 5.64, respectively. Negative charge residues are presented in red and positive charge residues in blue, the color intensity represents how exposed the ion group is. The isoelectric point was calculated using the tool expasy, PDB: 6XOK (TLL), 3TGL (RML), 4K5Q (CALB), and the structure of the Lecitase was built via homology using phyre2 (http://www.sbg.bio.ic.ac.uk/phyre2/html/page.cgi?id=index accessed on 15 October 2023), taking into account that it is a chimeric enzyme composed of the sequence from residues 1–284 of TLL and 285–339 of *F. oxysporum* [44]. Figure S2: Biodiesel yield (% *w*/*w*) of the different enzymes immobilized in Lewatit^®^ VP OC 1600 and the commercial reference for TLL Lipozyme^®^ TL IM, using 3.1:1 ethanol: palm olein, 37 °C, 1700 rpm [9]. Figure S3: EE yield produced by TLL immobilized in different type of supports. Reaction conditions: 3.1:1 ethanol: palm olein, 37 °C, 1700 rpm [9]. The abbreviations from left to right correspond to: Novo^®^ (Novozyme^®^ 435), Lipo^®^ (Lipozyme^®^ TL IM), SP (Sulfopropyl Sepharose^®^), Q (Q-Sepharose^®^), DexSO_4_ (Dextran Sulfate agarose), PEI (Polyethyleneimine-agarose), NK (Nekrolith^®^), VPOC (Lewatit^®^ VP OC 1600), MPSP (Lewatit^®^ MPSP112H), MP800 (Lewatit^®^ MP800) and PL (Purolite^®^). Figure S4: Representative cluster using AutodockVina between open TLL (blue) and CTAB (green/red). The catalytic triad (red) and the residues closest to the detergent (orange) are highlighted. Also, using Discovery Studio the interactions in 2D are here represented: van der Waals (green light), conventional hydrogen bond (green) and unfavorable interactions (red). Figure S5. Representative cluster using AutodockVina between open TLL (blue) and SDS (green). The catalytic triad (red) and the residues closest to the detergent (orange) are highlighted. Also, using Discovery Studio the interactions in 2D are represented here: van der Waals (green light) and alkyl, Pi-Sulfur (orange) and Pi-Alkyl (pink). Figure S6. Representative cluster using AutodockVina between open TLL (blue) and p-NFB (green). The catalytic triad (red), the residues closest to the substrate (orange) and the polar interactions between groups (yellow dotted line) are highlighted. Also, using Discovery Studio the interactions in 2D are represented here: van der Waals (green light), conventional hydrogen bond (green) and alkyl and Pi-Alkyl (pink). Figure S7. Representative cluster TLL (PDB: 6XOK) open (blue) with CTAB (left) coupling with p-NPB substrate (right). The catalytic triad (red) and the residues closest to the surfactant (orange) are highlighted. Here it is observed that for both cases, the hydrophobic end is responsible for interacting with the active site domain, calculated using AutoDock Vina (http://pyrx.sourceforge.net accessed on 15 October 2023), The Scripps Research Institute [35]. Also, using Discovery Studio the interactions between cluster enzyme-CTAB with p-NPB in 2D are represented here: van der Waals (green light) and alkyl and Pi-Alkyl (pink). Figure S8. Hydrolytic activity of TLL derivative Q-Sepharose^®^ obtained without additives, adding to the reaction medium different quantities CTAB, SDS, PEI or CMC. Activity of the derivative in the absence of modifiers = 1 (0.09 UI). The union between points is only to facilitate the visualization of the graph. Figure S9. Hydrolytic activity of TLL derivative Q-Sepharose^®^ obtained in the presence of CTAB, by adding to the reaction medium different quantities of CTAB, SDS, PEI or CMC. Activity of the derivative in the absence of modifiers = 1 (0.048 UI). The union between points is only to facilitate the visualization of the graph. Figure S10. Time-course tracking of %EE palm production for Q-SDS-TLL derivatives with enzyme loads between 1 mg TLL/g support and 25 mg TLL/g support. Reaction at 37 °C at 1700 rpm using 40 mg by mass of derivative. Table S1: Percentage loss of enzyme activity (%) with respect to the initial activity as a function of pH in 20 h, free enzyme. Table S2: EE production (%) using Q-TLL and Q-CTAB-TLL derivatives with immobilized enzyme loading of 1 mg/g and 10 mg/g. Table S3: EE production (%) using refined and used oil, by derivatives based on glyoxyl-agarose support modified with quaternary amino groups (GxGT), comparing when SDS is used and not used in the immobilization process.

## Abbreviations

BCA	Bicinchoninic acid
BSA	Bovine serum albumin
CALB	*Candida antarctica B*
CMC	Carboxy-methylcellulose
CTAB	Hexadecyltrimethylammonium bromide
DexSO_4_	Dextran Sulfate agarose (−)
EE	Ethyl esters fatty acids
MP800	Lewatit^®^ MP800
MPSP	Lewatit^®^ MPSP112H
NK	Nekrolith^®^(+)
OC	Octyl-Sepharose^®^ *
PEI	Polyethylenimine
PL^®^	Purolite^®^ ECR1604
*p*-NPB	*p*-nitrophenyl butyrate
Q	Q-Sepharose^®^ (+)
RML	*Rhizomucor miehei*
SDS	Sodium dodecyl sulfate
SP	Sulfopropyl Sepharose^®^ (−)
TLL	*Thermomyces lanuginosus lipase*
TX	Triton^®^ X-100
VPOC	Lewatit^®^ VPOC1600
