# Supplementary material for: Biocatalysts Based on Immobilized Lipases for the Production of Fatty Acid Ethyl Esters: Enhancement of Activity through Ionic Additives and Ion Exchange Supports
Source: BioTech (Basel). 2023 Dec 18;12(4):67. doi: 10.3390/biotech12040067 (PMC10742180; doi:10.3390/biotech12040067)
Supplement: Supplementary file 1 [file biotech-12-00067-s001.zip › biotech-2621287-supplementary.pdf]

Supplementary material

Table S1. Percentage loss of enzyme activity (%) with respect to the initial activity as a function of pH after 20 h (free enzyme).

TLL		CALB		RML		*Lecitase®*	
**pH**	%	pH	%	pH	%	pH	%
**3.5**	62.0	3.5	39.0	3.5	43.4	3.5	63.9
**8.0**	8.62	8.0	12.9	8.0	6.15	8.0	3.5
**8.5**	22.2	9.5	13.5	-	-	8.5	31.7
**-**	-	10.0	22.7	-	-	-	-

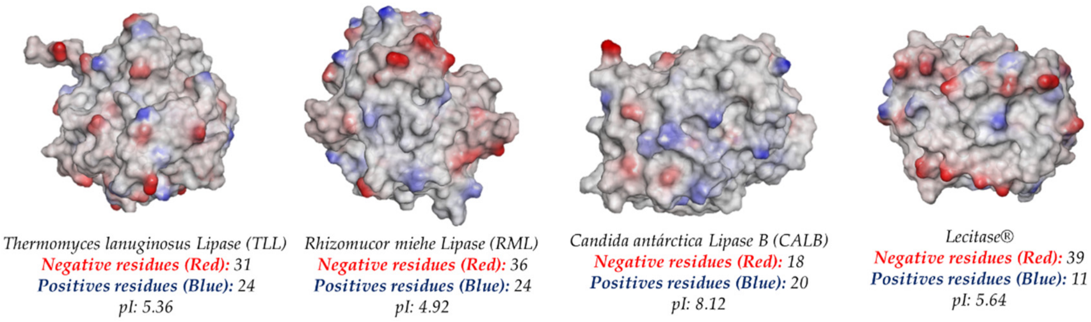

Figure S1. Representation of surface load density of lipases from *Thermomyces lanuginosus* (TLL), *Rhizomucor miehei* (RML), *Candida antarctica B* (CALB) y Lecitase®, the isoelectric points are 5.36, 4.92, 8.12 and 5.64, respectively. Negative charge residues are presented in red and positive charge residues in blue, the color intensity represents how exposed the ion group is. The isoelectric point was calculated using the tool expasy, PDB: 6XOK (TLL), 3TGL (RML), 4K5Q (CALB) and the for the structure of the Lecitase was built by homology using phyre2 (http://www.sbg.bio.ic.ac.uk/phyre2/html/page.cgi?id=index), taking into account that it is a chimeric enzyme composed of the sequence from residues 1-284 of TLL and 285-339 of *F. oxysporum* [44].

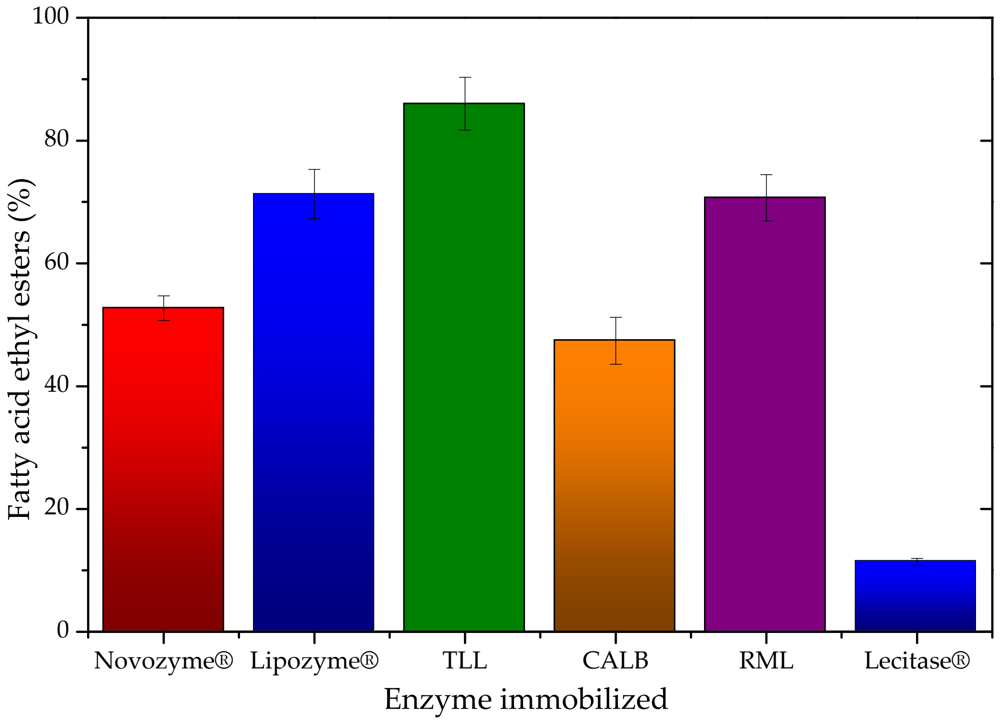

Figure S2. Biodiesel yield (% w/w) of the different enzymes immobilized in Lewatit® VP OC 1600 and the commercial reference for TLL Lipozyme® TL IM, using 3.1:1 ethanol: palm olein, 37 °C, 1700 rpm [9].

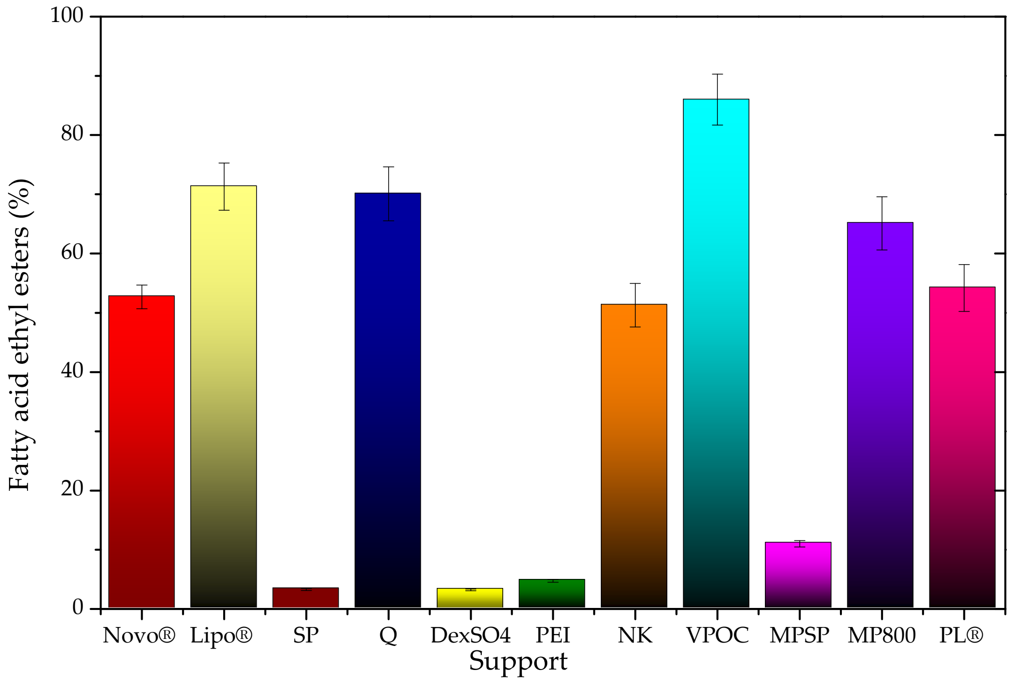

Figure S3. EE yield produced by TLL immobilized in different type of supports. Reaction conditions: 3.1:1 ethanol: palm olein, 37 °C, 1700 rpm [9]. The abbreviations from left to right correspond to: Novo® (Novozyme® 435), Lipo® (Lipozyme® TL IM), SP (Sulfopropyl Sepharose®), Q (Q-Sepharose®), $DexSO_4$ (Dextran Sulfate agarose), PEI (Polyethyleneimine-agarose), NK (Nekrolith®), VPOC (Lewatit® VP OC 1600), MPSP (Lewatit® MPSP112H), MP800 (Lewatit® MP800) and PL (Purolite®).

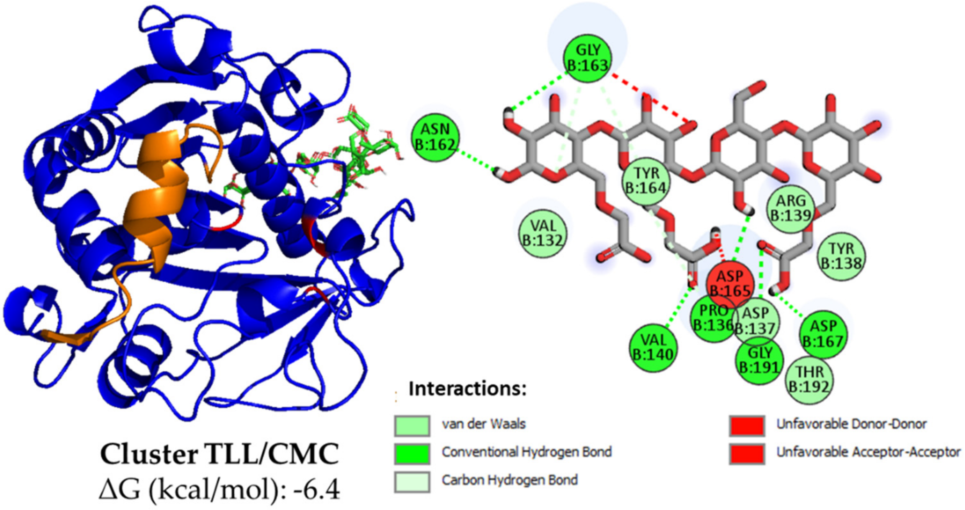

Figure S4. Representative cluster using AutodockVina between open TLL (blue) and CTAB (green/red). The catalytic triad (red) and the residues closest to the detergent (orange) are highlighted. Also, using Discovery Studio here are represented the interactions in 2D: van der Waals (green light), conventional hydrogen bond (green) and unfavorable interactions (red).

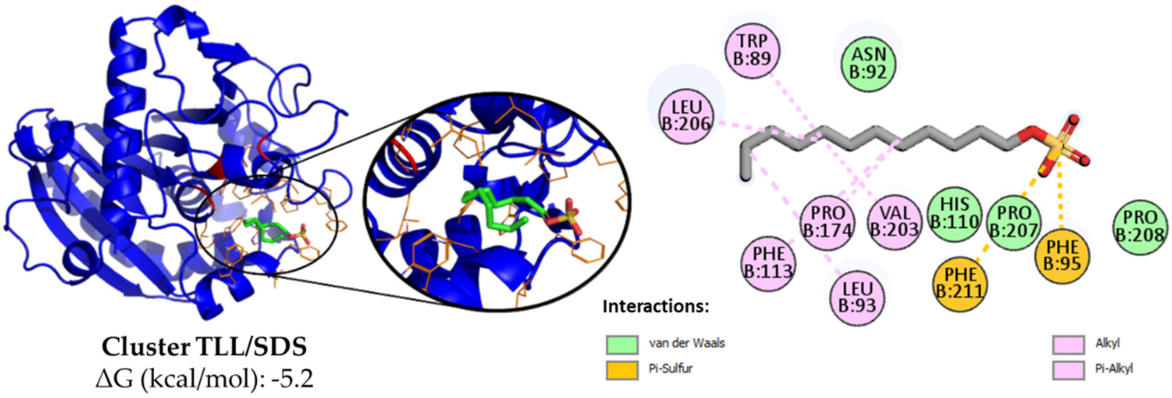

Figure S5. Representative cluster using AutodockVina between open TLL (blue) and SDS (green). The catalytic triad (red) and the residues closest to the detergent (orange) are highlighted. Also, using Discovery Studio here are represented the interactions in 2D: van der Waals (green light) and alkyl, Pi-Sulfur (orange) and Pi-Alkyl (pink).

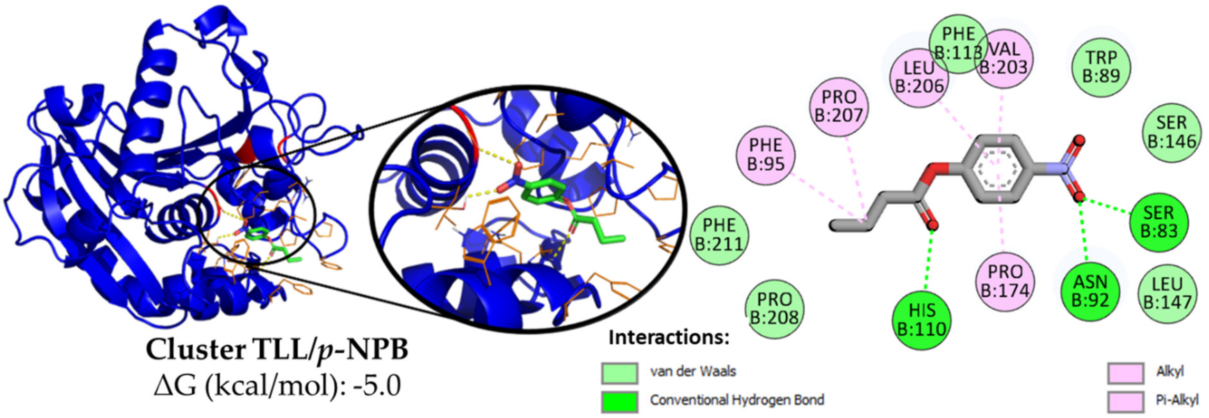

Figure S6. Representative cluster using AutodockVina between open TLL (blue) and *p*-NPB (green). The catalytic triad (red), the residues closest to the substrate (orange) and the polar interactions between groups (yellow dotted line) are highlighted. Also, using Discovery Studio here are represented the interactions in 2D: van der Waals (green light), conventional hydrogen bond (green) and alkyl and Pi-Alkyl (pink).

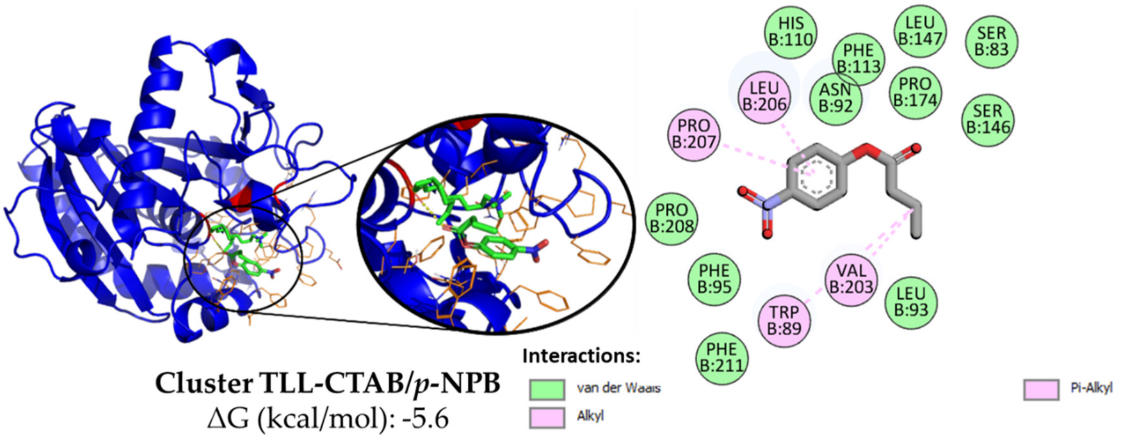

Figure S7. Representative cluster TLL (PDB: 6XOK) open (blue) with CTAB (left) coupling with *p*-NPB substrate (right). The catalytic triad (red) and the residues closest to the surfactant (orange) are highlighted. Here it is observed that for both cases, the hydrophobic end is responsible for interacting with the active site domain, calculated with AutoDock Vina (http://pyrx.sourceforge.net/), The Scripps Research Institute [35]. Also, using Discovery Studio here are represented the interactions between cluster enzyme-CTAB with *p*-NPB in 2D: van der Waals (green light) and alkyl and Pi-Alkyl (pink).

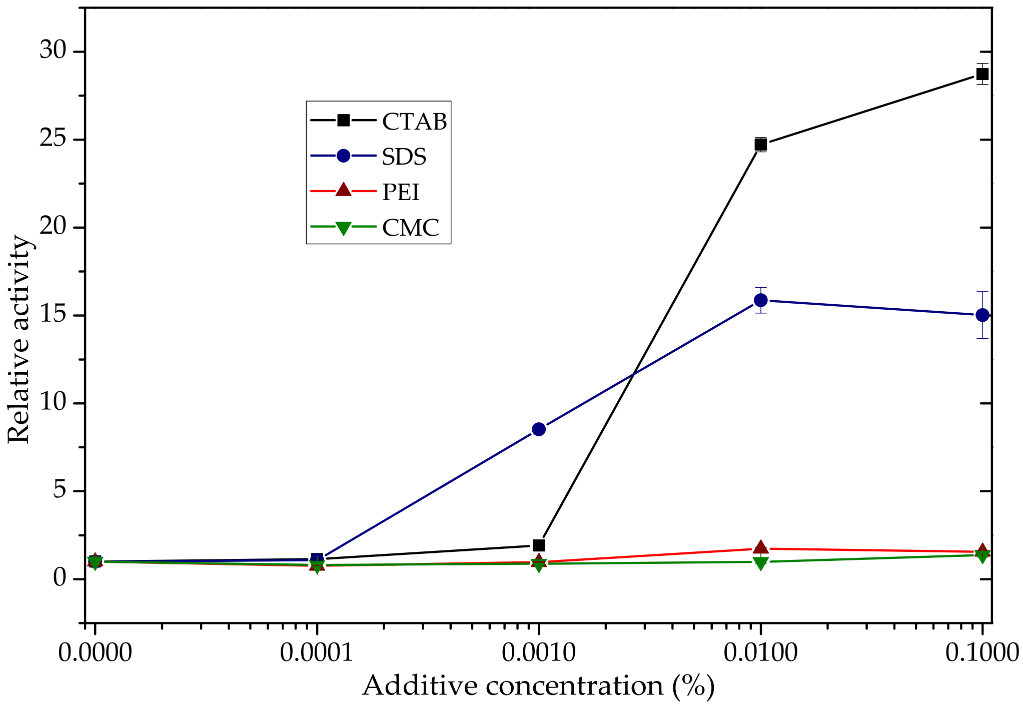

Figure S8. Hydrolytic activity of TLL derivative Q-Sepharose® obtained without additives, adding to the reaction medium different quantities CTAB, SDS, PEI or CMC. Activity of the derivative in the absence of modifiers = 1 (0.09 UI). The union between points is only to facilitate the visualization of the graph.

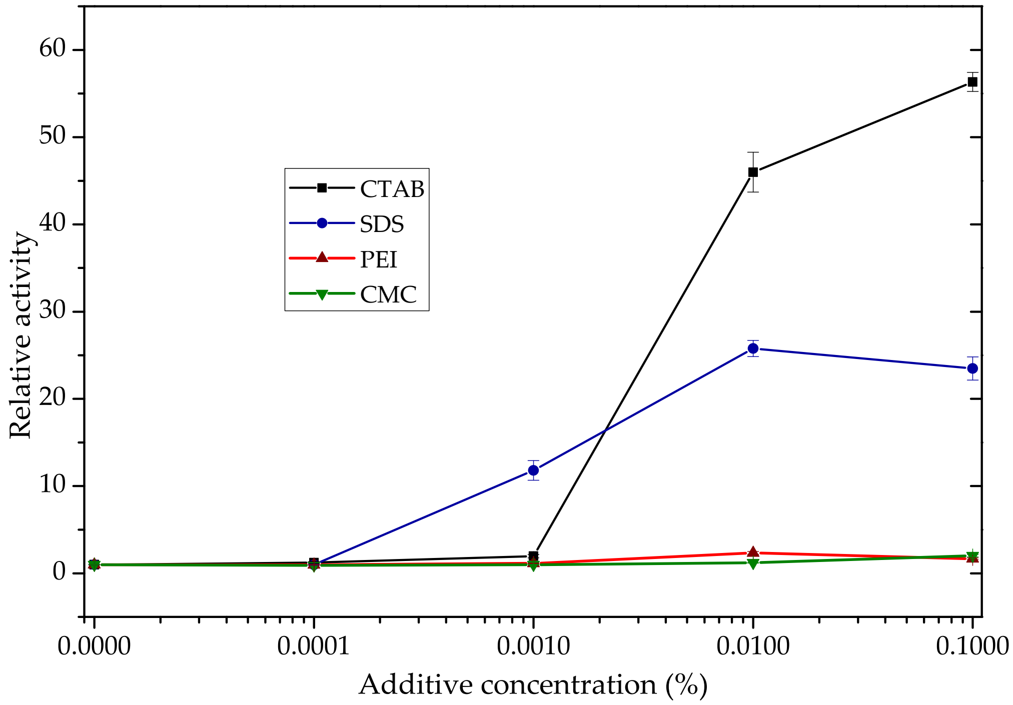

Figure S9. Hydrolytic activity of TLL derivative Q-Sepharose® obtained in the presence of CTAB, adding to the reaction medium different quantities of CTAB, SDS, PEI or CMC. Activity of the derivative in the absence of modifiers = 1 (0.048 UI). The union between points is only to facilitate the visualization of the graph.

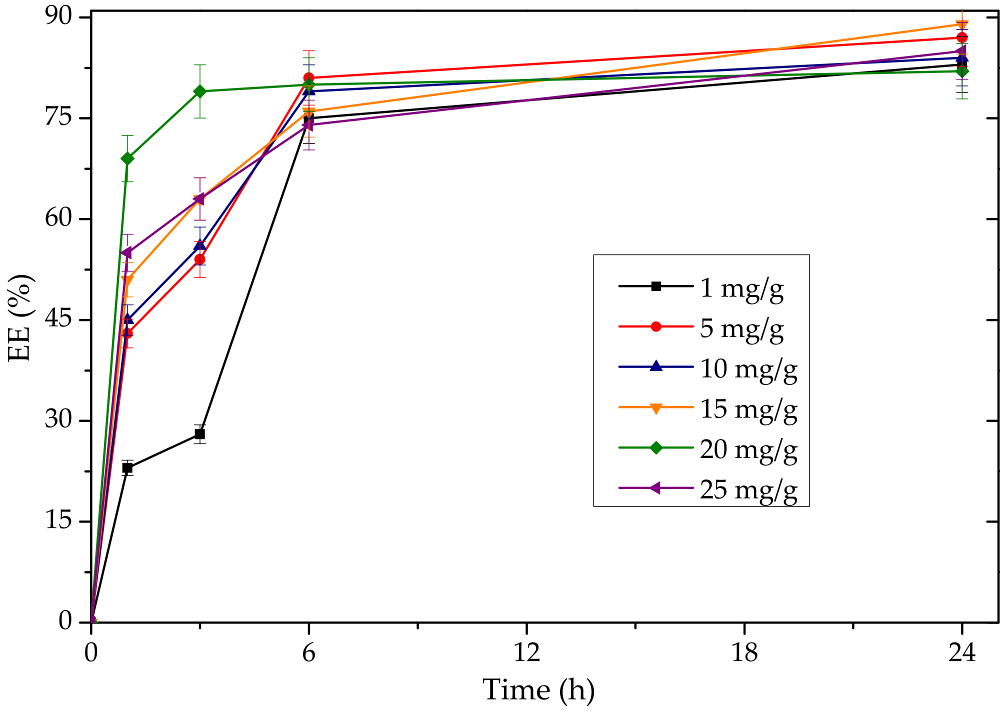

Figure S10. Time-course tracking of %EE palm production for Q-SDS-TLL derivatives with enzyme loads between 1 mg TLL/g support – 25 mg TLL/g support. Reaction at 37 °C at 1700 rpm using 40 mg by mass of derivative.

Table S2. EE production (%) using Q-TLL and Q-CTAB-TLL derivatives with immobilized enzyme loading of 1 mg/g and 10 mg/g.

	**Q-TLL**		**Q-CTAB-TLL**	
**Time (h)**	1 mg/g	10 mg/g	1 mg/g	10 mg/g
1	5.8 ± 4.5	7.2 ± 1.3	2.6 ± 0.3	9.0 ± 3.4
6	15.5 ± 2.5	21.4 ± 3.7	6.3 ± 1.6	18.1 ± 4.2
24	16.6 ± 0.7	31.0 ± 4.3	14.9 ± 2.5	27.2 ± 2.9

Table S3. EE production (%) using refined and used oil, by derivatives based on the glyoxyl-agarose support having quaternary amino groups (GxGT). Here it is shown the effect of the presence or absence of SDS during immobilization which also reflects the improvement observed for the Q-Sepharose® type derivatives.

	**Refined palm oil**		**Used oil**	
**Time (h)**	Gx-GT-TLL	Gx-GT-SDS-TLL	Gx-GT-TLL	Gx-GT-SDS-TLL
1	9.8 ± 2.5	28.8 ± 1.3	7.0 ± 0.3	12.3 ± 3.4
6	19.3 ± 1.4	51.4 ± 1.3	17.3 ± 2.2	35.1 ± 1.9
24	38.3 ± 6.0	62.0 ± 3.6	34.6 ± 3.6	64.2 ± 2.9
